# Evaluation of β-Catenin Inhibition of Axitinib and Nitazoxanide in Human Monocyte-Derived Dendritic Cells

**DOI:** 10.3390/biomedicines9080949

**Published:** 2021-08-03

**Authors:** Waqas Azeem, Ragnhild Maukon Bakke, Benjamin Gabriel, Silke Appel, Anne Margrete Øyan, Karl-Henning Kalland

**Affiliations:** 1 Department of Microbiology, Haukeland University Hospital, Helse Bergen, NO-5021 Bergen, Norway; Ragnhild.Bakke@uib.no (R.M.B.); Benjamin.Gabriel@uib.no (B.G.); 2 Department of Clinical Science, University of Bergen, NO-5021 Bergen, Norway; Silke.Appel@uib.no (S.A.); Anne.Oyan@uib.no (A.M.Ø.); 3 Broegelmann Research Laboratory, University of Bergen, NO-5021 Bergen, Norway; 4 Department of Immunology and Transfusion Medicine, Haukeland University Hospital, Helse Bergen, NO-5021 Bergen, Norway; 5 Norway Centre for Cancer Biomarkers, (CCBIO), University of Bergen, NO-5021 Bergen, Norway

**Keywords:** monocyte-derived dendritic cell, beta-catenin, ICG-001, axitinib, nitazoxanide

## Abstract

Modulation of β-catenin signaling has attractive therapeutic potential in cancer immunotherapy. Several studies have found that β-catenin can mediate immune evasion in cancer and promote anti-inflammatory features of antigen-presenting dendritic cells. Many small molecular compounds that inhibit Wnt/β-catenin signaling are currently in clinical development, but none have entered routine clinical use. New inhibitors of β-catenin signaling are consequently desirable. Here, we have tested, in monocyte-derived dendritic cells, the effects of two small molecular compounds, axitinib and nitazoxanide, that previously have been discovered to inhibit β-catenin signaling in colon cancer cells. Immature and lipopolysaccharide-matured dendritic cells prepared from healthy blood donor buffy coats were stimulated with 6-bromoindirubin-3′-oxime (6-BIO) to boost basal β-catenin activity, and the effects of axitinib and nitazoxanide were compared with the commercial β-catenin inhibitor ICG-001. Assays, including genome-wide RNA-sequencing, indicated that neither axitinib nor nitazoxanide demonstrated considerable β-catenin inhibition. Both compounds were found to be less toxic to monocyte-derived dendritic cells than either 6-BIO or ICG-001. Axitinib stimulated several aspects of dendritic cell function, such as IL12-p70 secretion, and counteracted IL-10 secretion, according to the present study. However, neither axitinib nor nitazoxanide were found to be efficient β-catenin inhibitors in monocyte-derived dendritic cells.

## 1. Introduction

Aberrant β-catenin activation has been implicated in cancer stem cell proliferation [1,2], cancer progression [3], and intrinsic cancer cell immune evasion [4,5,6,7]. Additionally, β-catenin has emerged as an important regulator of the immune system [8,9,10,11]. In dendritic cells, β-catenin signaling favors tolerogenic features and inhibits pro-inflammatory features [12]. It is conceivable that β-catenin inhibition could have both anti-oncogenic and immunotherapeutic effects that could provide synergetic cancer therapy. While many compounds have been found to inhibit various steps of Wnt/β-catenin signaling, several of which are in clinical trials, none of these compounds have been approved for clinical standard of care yet [9,13,14]. New, small molecular β-catenin inhibitors are thus in great demand.

Using a repurposing screening strategy, our group has identified that the small molecular compounds axitinib and nitazoxanide each target β-catenin signaling in colon cancer cells in which β-catenin was constitutively over-activated [15,16,17]. The potential advantage of the two compounds is that both axitinib and nitazoxanide have already been approved for therapeutic use in humans. Axitinib is a receptor tyrosine kinase inhibitor of vascular endothelial cell growth factor receptors (VEGFR) 1, 2, and 3, platelet-derived growth factor, and c-Kit, and approved as an oral medication for selected cancers [18]. It was separately found to target the E3-ligase SHPRH with increased nuclear ubiquitination and degradation of β-catenin as the secondary result [15]. Nitazoxanide is an antiparasitic agent [19] and has been found to target the enzyme PADI2 with increased citrullination and degradation of β-catenin [17].

In cancer therapy it is not sufficient that a drug can target cancer cells; it must additionally be taken into consideration how that compound impacts other cells of the body. During cancer drug development it is critical to examine the consequence of new drugs on normal body cells and functions, and how these compounds may affect immune cells. Here, we wanted to examine the effect of axitinib and nitazoxanide in monocyte-derived dendritic cells (moDCs). DCs are the most potent antigen-presenting cells (APCs) of the immune system, and play a vital role in initiating the adaptive immune response as well as maintaining tolerance to self-antigens [20]. As immature cells, they continually search their environment for antigens, while as mature cells they migrate to the lymph nodes and present processed antigens to T lymphocytes on their MHC molecules. Due to their role in development of both immunity and tolerance, DC-based immunotherapies have been extensively developed, but still have a limited documented response rate [21].

Lipopolysaccharide (LPS) is a major membrane component of Gram-negative bacteria. LPS is commonly used experimentally to induce rapid maturation of immature DCs following its recognition by the DC Toll-like receptor 4 (TLR4). We have previously reported that there is basal β-catenin activation in LPS-matured moDCs, and β-catenin activity can be augmented using the GSK-3β inhibitor 6-BIO. Treatment of moDCs with either 6-BIO or the β-catenin inhibitor ICG-001 showed that these two compounds competed dose-dependently regarding anti- and pro-inflammatory features, respectively. The 6-BIO increased secretion of the anti-inflammatory cytokine IL-10 and decreased the secretion of the pro-inflammatory cytokine IL12. ICG-001 treatment had the opposite and competing effect [12]. This is consequently an attractive model for the test of potential β-catenin inhibitors in dendritic cells.

## 2. Materials and Methods

### 2.1. Cell Cultures

Peripheral blood mononuclear cells (PBMC) were collected through Ficoll-Lymphoprep (Cat.no. 1114545, Axis-Shield, Dundee, United Kingdom) from buffy coats of healthy blood donors at Haukeland University Hospital. Samples were anonymized according to the Norwegian Regional Ethical Committee approval #64205. Monocytes were then isolated using the Monocyte Isolation Kit II human (Cat.no. 130-091-153, Miltenyi Biotec, Bergisch Gladbach, North Rhine-Westphalia, Germany) or Pan Monocyte Isolation Kit (Cat.no. 130-096537, Miltenyi Biotec) supplemented with CD61 microbeads (Cat.no. 130-051-101, Miltenyi Biotec) and LD columns (Cat.no. 130-042-901, Miltenyi Biotec) or LS columns (Cat.no. 130-042-401, Miltenyi Biotec). The untouched monocytes were cultured in RP10 medium (RPMI 1640, Cat.no. 12-702F, LONZA, Bioscience, Basel, Switzerland), supplemented with 10% FCS (Cat.no. F7524, Sigma Aldrich, St. Louis, MO, USA), 1% Penicillin-Streptomycin (Cat.no. DE17-603E, LONZA) and L-Glutamate (Cat.no. BE14-605E, LONZA)) or CellGenix GMP DC medium (Cat.no. 20801-0500, CellGenix, Freiburg im Breisgau, Germany), seeded at cell densities of 1.5 × 10^6^ per 3 mL/well in 6-well plates (Cat.no. 92006, TPP Techno Plastic Products AG, Trasadingen, Switzerland), or 0.75 × 10^6^ per 1.5 mL/well in 12-well plates (Cat.no. 92412, TPP Techno Plastic Products AG). Media were supplemented with 20 ng/mL IL-4 (Cat.no. 11340047, Immunotools, Friesoythe, Germany) and 100 ng/mL GM-CSF (Cat.no. 11343128, Immunotools). The same concentrations of IL-4 and GM-CSF were additionally added on day 3 of culture. On day 4 were added, if not stated otherwise, 0.5 μM–1 μM 6-bromoindirubin-3-oxime (6-BIO, Cat.no. S7198, Selleckchem, Houston, TX, USA), 0.5 μM–8 μM ICG-001 (Cat.no. S2662, Selleckchem), 2.5 μM–10 μM axitinib (Cat.no. S1005, Selleckchem), 5 μM–20 μM nitazoxanide (Cat.no. 1627, Selleckchem), or DMSO (solvent of all 10 mM stock solutions of 6-BIO, ICG-001, axitinib, and nitzoxanide) vehicle as control, and 1 h later, 100 ng/mL LPS (Cat.no. L4391, Sigma Aldrich) was added as maturation stimulus for 23 h before harvest.

### 2.2. MTS Assay (CellTiter 96 Aqueous One Solution Cell Proliferation Assay)

Monocytes were seeded in 96-well plates, with 5 × 10^4^ cells per 100 μL RP10 medium per well. After 4 days of differentiation and 24 hr of treatment with compounds in various concentrations, 10 μL MTS reagent (CellTiter 96^®^ Aqueous One Solution Cell Proliferation Assay, Promega, Madison, WI, USA), Cat.no. G3582) was added to each well and incubated for 3 hrs at 37 °C. Absorbance at 490 nm was read with Synergy H1 Hybrid Multi-Mode Reader and analyzed by using Gen5 2.00.18 software (BioTek Instruments, Winooski, VT, USA).

### 2.3. Western Blots

Whole-cell lysate was prepared using RIPA buffer (Cat.no. ab156034, Lot: K9419, abcam, Cambridge, United Kingdom). A total of 20 μg protein was added to each well in a Bolt™ 4–12% Bis-Tris Plus gel (1.0 mm, 10 well, NuPAGE, NW04120BOX) and blotted on to Amersham Hybond P 0.45 μm PVDF membrane (Cat.no. 10600069, GE Healthcare, Chicago, IL, USA). For protein size, the Precision Plus Protein™ Kaleidoscope Marker (Cat.no. 161-0375, Biorad, Hercules, CA, USA) was added to one well. Membranes were blocked with 5% skim milk in PBS-T (0.1% Tween20 in 1× phosphate-buffered saline, PBS) for one hr. β-catenin was visualized with primary antibody anti-β-catenin (Cat.no. ab16051, abcam) in dilution 1:2000, while GAPDH (Cat.no. MA1-16757, Invitrogen, Waltham, MA, USA) in 1:10000 was used for normalization. Membranes were washed in PBS-T and incubated for one hr with anti-mouse HRP conjugate (Cat.no. 170-5017, BioRad) or IgG HRP-linked whole antibody (ECL™, donkey, Cat.no. GENA935, Merck, Kenilworth, IL, USA) as secondary antibodies, both 1:5000. Additionally, 5% skim milk in PBS-T was used for antibody dilution. Proteins were visualized by chemiluminescence and captured with Quantity One software.

### 2.4. Indirect Immunofluorescence Microscopy

Slides with cells were obtained by cytospin of 200 μL cell suspension (0.5 × 10^5^ DCs/mL). The slides were then kept in 100% methanol to fix on ice or at 4 °C for 15 min or longer until immunofluorescence (IF) was performed, washed in PBS, and blocked with 0.5% bovine serum albumin (BSA) in PBS for 1 h at room temperature. The primary antibody used was anti-β-catenin (Cat.no. ab16051, abcam) at 1:1000 dilution, and FITC-conjugated Pierce^TM^ goat anti-rabbit IgG (H + L) secondary antibody (Cat.no. 31635; Thermo Fisher Scientific, Waltham, MA, USA) was used at 1:50 dilution. Cells grown on coverslips were mounted on glass slides in Slowfade^TM^ Gold Antifade Mountant with DAPI (Cat.no. S36939, Thermo Fisher Scientific). After staining, the slides were analyzed using the BioTek^®^ Cell Imaging Multi-Mode Reader Cytation 5 for GFP and DAPI.

### 2.5. Flow Cytometry Analyses

A panel of nine antibodies was used for phenotyping of the moDCs using a BD Fortessa flow cytometer as described previously [12]. The panel included antihuman antibodies against CD14-FITC (18D11, Cat.no. 21620143, Immunotools), CD1a-PE (HI149, Cat.no. 21270014, Immunotools), HLA-DR-Horizon V500 (G46-6, Cat.no. 561224, BD Biosciences), CCR7 Brilliant Violet 421 (G043H7, Cat.no. 353208, Biolegend), CD80-Brilliant Violet 605 (2D10, Cat.no. 305225, Biolegend, San Diego, CA, USA) CD86-AlexaFluor 647 (IT2.2, Cat.no. 305416, Biolegend), CD83-BD Horizon PE-CF594 (HB15e, Cat.no. 562631, BD Biosciences, San Jose, CA, USA), CD273 PE-Cy7 (PD-L1) (M1H18, Cat.no. 46-5888-42, eBioscience, Waltham, MA, USA), and CD274 PerCP-Cy5.5 (PD-L2) (B7-H1, Cat.no. 46-5983-42, eBioscience). All flow cytometric analyses were performed using FlowJo software (Version 10, FlowJo, LLC, Ashland, OR, USA). A representative gating strategy is shown in Supplementary Figure S3.

### 2.6. Enzyme-Linked Immunosorbent Assay (ELISA)

Cell culture supernatants were analyzed for cytokines with Human IL-10 ELISA MAX™ Deluxe kit (Cat.no. 430604, BioLegend), IL-10 Human Uncoated ELISA Kit (Cat.no. 88-7106-88, Invitrogen), IL-12p70 Human Uncoated ELISA Kit (Cat.no. 88-7126-88, Invitrogen), and Human VEGF DuoSet ELISA (Cat.no DY293B, R&D Systems, Abingdon, United Kingdom). The absorbance was measured with Synergy H1 Hybrid Multi-Mode Reader and analyzed by using Gen5 2.00.18 software (BioTek^®^).

### 2.7. Mixed Leukocyte Reaction (MLR)

Allogeneic MLR was performed as described previously [22] by adding 2 × 10^5^ monocyte-depleted allogeneic PBMCs labeled with CFSE, using Vybrant^TM^ CFDA SE Cell Tracer Kit (Cat. no. V12883, Invitrogen), to 5 × 10^4^ treated moDCs for 5 days in X-Vivo 20 medium (Cat. no. 04-448Q; Lonza) supplemented with 50 U/mL of IL-2 (Cat. no. 11340023; Immunotools) and 10 ng/mL of IL-7 (Cat. no. 11340073; Immunotools). CD3/CD28-beads (Cat.no. 11161D, Gibco, Waltham, MA, USA) were used as positive control and CFSE-stained lymphocytes only as negative control. The cells were harvested on day 5 and analyzed on an Acurri C6 flow cytometer (BD Biosciences). 

### 2.8. Endocytosis Assay

The endocytic capacity of the generated moDC populations was determined by fluorescent dextran uptake. A total of 5 × 10^4^ moDCs were incubated with 0.25 mg/mL fluorescein isothiocyanate (FITC)-labeled dextran (molecular weight 40,000; Cat.no. D1844, Molecular Probes, Thermofisher Scientific) in a 96-well plate at 37 °C for one hr. As a control, 5 × 10^4^ moDCs were precooled to 4 °C before the incubation with FITC-dextran at 4 °C for one hr. After incubation, cells were washed four times with 0.5% BSA in PBS. Cells were analyzed on an Accuri C6 flow cytometer, and further analysis was performed using FlowJo V10 software (FlowJo, LLC). The results obtained represent five independent experiments.

### 2.9. Chemotaxis Assay

For the analysis of chemotactic activity, 5 × 10^4^ moDCs were added to the upper chamber and 100 ng/mL CCL19 (Cat. no. 11343240; Immunotools) chemoattractant was added to the bottom chamber of a 96- trans-well plate with 8 μm pore size polyester membrane (Cat. no. 3384; Corning, New York, USA). After the incubation for four hrs at 37 °C, the transmigrated moDCs were collected from the bottom chamber and counted by CASY™ cell counter (Schärfe System, Reutlingen, Germany). The results obtained represent five independent experiments.

### 2.10. RNA-Sequencing and Analyses

All sequencing was conducted at Qiagen Genomic Services, Hilden, Germany as previously described [12]. The library preparation was carried out using TruSeq^®^ Stranded mRNA Sample preparation kit (Cat.no. 20020594, Illumina Inc., San Diego, CA, USA). The starting material (500 ng) of total RNA was mRNA enriched using the oligodT bead system. The isolated mRNA was subsequently enzymatically fragmented. Then, first-strand synthesis and second-strand synthesis were performed, and the double-stranded cDNA was purified (AMPure XP, Cat.no. A63881, Beckman Coulter, Brea, CA, USA). The cDNA was end-repaired, 3′ adenylated, and Illumina sequencing adaptors ligated onto the fragments ends, and the library was purified (AMPure XP). The mRNA-stranded libraries were preamplified with PCR and purified (AMPure XP). The library’s size distribution was validated, and quality-inspected on a Bioanalyzer 2100 or BioAnalyzer 4200tape Station (Agilent Technologies, Santa Clara, CA, USA). High-quality libraries were pooled based on equimolar concentrations based on the Bioanalyzer Smear Analysis tool (Agilent Technologies). The library pool(s) were quantified using qPCR and optimal concentration of the library pool used to generate the clusters on the surface of a flow cell before sequencing on a NextSeq500 instrument (75 cycles) according to the manufacturer’s instructions (Illumina Inc.).

### 2.11. Software Tools Used for RNA-Seq Analysis

Sequencing and alignment and transcript quantification were performed at Qiagen using CLC Workbench. Raw read counts were reported back to our group and used as the input for the differential gene expression (DGE) analysis. This analysis was performed using the DESeq2 package (v1.28.1) available for R (v4.0.2) [23]. The analysis was performed using the Wald test statistic including a minimum effect size of 0.585 (log fold-change) and a significance level α of 0.1. Under these conditions, genes showing an adjusted *p*-value of <0.1 were considered significant. Gene Ontology (GO) enrichment analysis was supported by the R package topGO (v2.42.0) [24] using Fisher’s exact test.

### 2.12. Statistical Analysis

All data were analyzed using GraphPad Prism 9 (GraphPad software, San Diego, CA, USA) if not stated otherwise. Statistical significance of the difference was calculated using two-way analysis of variance (ANOVA) followed by Dunnett’s multiple comparisons test with 95% confidence interval. A value of *p* ≤ 0.05 was considered statistically significant.

## 3. Results

### 3.1. Compounds Affect the Metabolic Activity in Human moDCs

**Figure 1:**
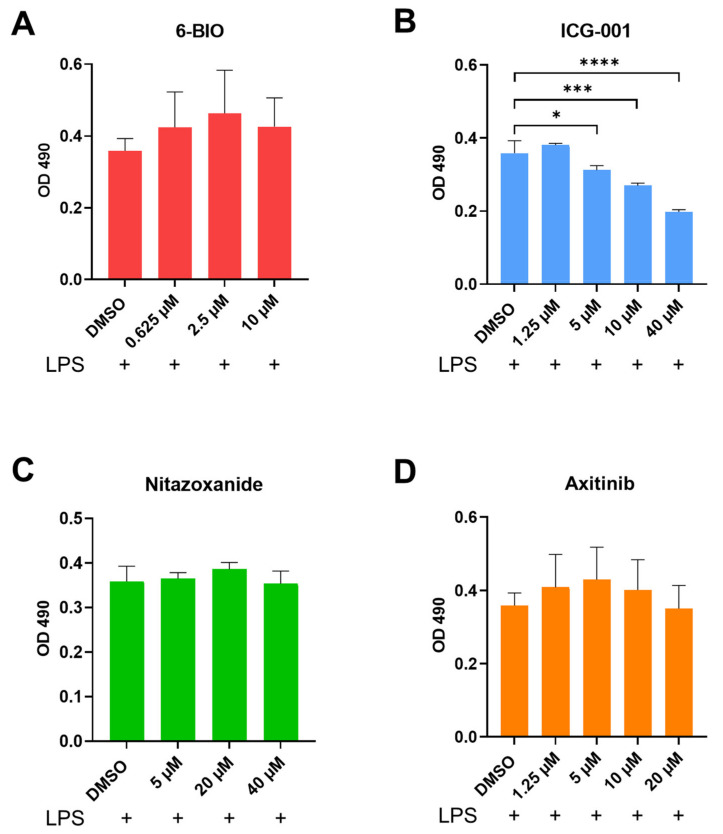
Results of MTS assays are shown as mean +/- SEM of the absorbance at OD 490 nm. The control is mature moDCs. Concentrations of the compounds in μM are shown along the x-axis. These graphs are based on 3 individual experiments. * *p* ≤ 0.05, *** *p* ≤ 0.001, **** *p* ≤ 0.0001 by using one-way ANOVA followed by Tukey’s multiple comparisons test with 95% confidence interval.

The MTS assay was used to measure the metabolic activity of moDCs and thus to determine the possible toxic effects of the compounds used. The 6-BIO showed no toxic effects up to 10 μM and was in the following experiments used at the lower concentrations up to 2 μM (Figure 1A). ICG-001 showed a toxic effect at 40 μM, but the effects were minimal at concentrations up to 10 μM (Figure 1B). The β-catenin inhibitors nitazoxanide and axitinib showed no toxic effects on moDCs up to 40 μM and 20 μM, respectively (Figure 1C,D). Thus, none of the four compounds had toxic effects at the concentrations used for the following experiments.

### 3.2. Dose-Dependent Effects of β-Catenin Modulators in LPS-Matured moDCs

**Figure 2:**
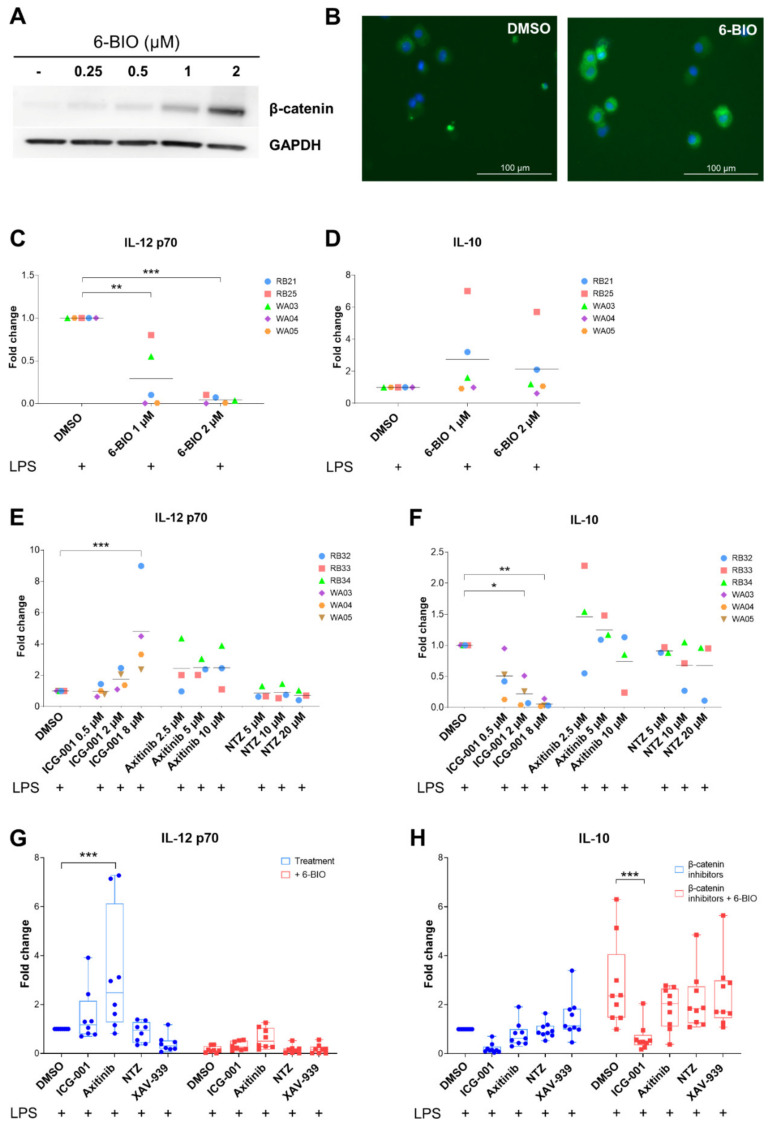
Immature moDCs obtained from healthy donors were treated with indicated concentrations of 6-BIO or β-catenin inhibitors with vehicle (DMSO) as control (type and concentrations as shown for (**A**,**C**–**F**) for 24 h with concomitant LPS for the last 23 h). (**A**) Western blot of whole-cell lysates. This blot is representative of 2 repeated experiments in healthy donors. (**B**) Immunofluorescence after cytospin. Cells were treated with DMSO or 2μM 6-BIO. This is representative of 2 repeated experiments in healthy donors. DAPI staining is blue and fluorescein-labeled β-catenin antibody is green. (**C**–**F**) The concentration of IL-12p70 and IL-10 in culture supernatants were measured with ELISA, shown as fold change compared to their respective mature moDCs control. (**G**,**H**) moDCs were treated with β-catenin inhibitors (4 µM ICG-001, 10 µM axitinib, 10 µM nitazoxanide (NTZ), 10 µM XAV-939) with or without 0.5 µM 6-BIO or DMSO (vehicle). The fold changes compared to respective DMSO control in culture supernatants were measured using ELISA. Corresponding concentrations in pg/mL are given in Supplementary Table S1. Each symbol represents a different donor, and lines represent the mean. Donors were anonymized and each buffy coat labeled by initials of experimenter (RB or WA) and consecutive buffy coat number. * *p* ≤ 0.05, ** *p* ≤ 0.01, *** *p* ≤ 0.001 by using two-way ANOVA followed by Dunnett’s multiple comparisons test with 95% confidence interval. For comparison tests, the cells treated with β-catenin inhibitors only or in combination of β-catenin inhibitors with 6-BIO were compared with DMSO or DMSO + 6-BIO controls, respectively.

We next tested small molecular compounds at a range of concentrations in LPS-matured moDCs in different assays, as exemplified in Figure 2. Dose-dependent increase of β-catenin at 6-BIO concentrations from 0.25 to 2 μM in LPS-matured moDC lysates according to Western blot is shown in Figure 2A, and increased single-cell accumulation of β-catenin in moDCs treated with 2 μM 6-BIO is visualized using indirect immunofluorescence analyses in Figure 2B. Dose-dependent decrease of IL-12p70 (Figure 2C) and, although not statistically significant, increase of IL-10 secretion (Figure 2D) to the cell culture supernatants are demonstrated for 1 and 2 μM 6-BIO using ELISA. Comparable dose–response effects on IL-12p70 and IL-10 secretion, induced by ICG-001 at 0.5–8 μM, axitinib at 2.5–10 μM, and nitazoxanide at 5 to 20 μM treatment of moDCs, were investigated and shown in Figure 2E,F, respectively. IL-12p70 secretion increased dose-dependently up to concentrations of 8 μM ICG-001 with an inverse dose-dependent decrease of IL-10 secretion at the same concentrations. Axitinib concentrations between 2.5 and 10 μM tended to increase IL-12p70 secretion (Figure 2E), although no dose-dependent effect was evident. By increasing the number of donors, it was shown that the increased IL12p70 secretion induced by 10 μM axitinib was clearly significant (Figure 2G, *p* < 0.001). Increased IL-10 levels were also associated with axitinib at 2.5 and 10 μM, but with a trend to dose-dependent decrease of IL-10 with increasing axitinib (Figure 2F,H). Clear dose-dependent effects were found neither on IL-12p70 secretion nor on IL-10 secretion at the range of tested nitazoxanide concentrations.

### 3.3. Effect of Axitinib and Nitazoxanide in Competition with 6-BIO on Cytokine Secretion

Based on the above findings and our previously published results [12], we went on to examine the ability of candidate β-catenin inhibitors to inhibit β-catenin activation induced by 0.5 μM 6-BIO. As shown in Figure 2G, 0.5 μM 6-BIO strongly reduced IL12-p70 secretion in LPS-matured moDCs compared to cells treated only with vehicle (DMSO). Both ICG-001 at 4 μM and axitinib at 10 μM partially counteracted the 6-BIO induced reduction (Figure 2G). Nitazoxanide at 10 μM was unable to counteract the 6-BIO effect on IL12-p70 secretion. The tankyrase and β-catenin inhibitor XAV-939 at 10 μM was one additional control that did not induce IL12-p70 and did not compete significantly with the 6-BIO inhibition of IL12-p70 secretion (Figure 2G). ELISA-quantified amounts of secreted cytokines differed between buffy coats of different healthy donors, as we also have reported previously [12]. The axitinib-induced increase of IL-12p70 and its counteraction of 6-BIO was statistically significant in contrast to the changes induced by either nitazoxanide or XAV-939. The other above trends did not, however, reach statistical significance (Figure 2G). 

The corresponding effects on IL-10 secretion and increased IL-10 secretion caused by 0.5 μM 6-BIO are shown in Figure 2H. ICG-001 at 4 μM significantly reduced IL-10 secretion in both vehicle-treated and 6-BIO-treated LPS-matured moDCs. On the contrary, neither axitinib at 10 μM, nor nitazoxanide at 10 μM nor XAV-939 at 10 μM were able to significantly counteract 6-BIO induced secretion of IL-10 (Figure 2H).

### 3.4. Effects of Axitinib and Nitazoxanide on moDC Surface Markers

**Figure 3:**
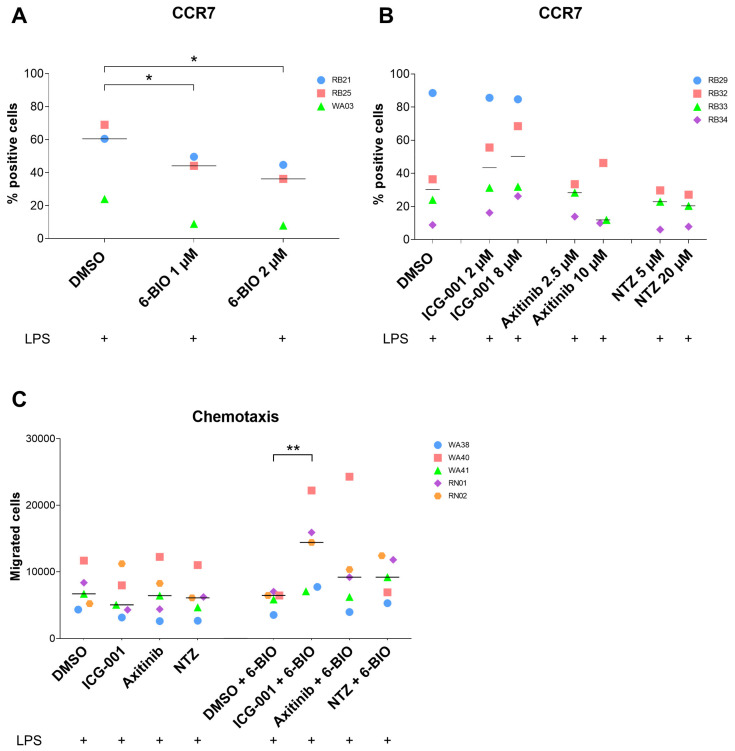
moDCs obtained from healthy donors were treated with 6-BIO or β-catenin inhibitors for 24 h. DMSO (vehicle) served as mature moDCs control and cells were treated with LPS for the last 23 h. (**A**,**B**) CCR7 cell surface marker was measured with flow cytometry and shown as change of percentage of positive cells following indicated compound treatments. (**C**) moDCs were treated with indicated β-catenin inhibitors (4 µM ICG-001,10 µM axitinib, 10 µM nitazoxanide (NTZ)) with or without 0.5 µM 6-BIO or DMSO (vehicle). A total of 50,000 cells were added to the upper chamber of an 8 μm trans-well 96-well plate and incubated for 4 h at 37 °C to migrate towards CCL19 (100 ng/mL) in the lower chamber. The migrated cells were counted using a CASY™ cell counter. Each symbol represents a different donor, and lines represent the median. Donors were anonymized and each buffy coat labeled by initials of experimenter (RB/RN or WA) and consecutive buffy coat number. * *p* ≤ 0.05, ** *p* ≤ 0.01 by using two-way ANOVA followed by Dunnett’s multiple comparisons test with 95% confidence interval. For comparison tests, the cells treated either with β-catenin inhibitors only or with combination between β-catenin inhibitors and 6-BIO were compared with DMSO or DMSO + 6-BIO controls, respectively.

In parallel to the measurements of cytokine secretion, we examined the expression of monocyte-derived dendritic cell membrane markers. We found that treatment with 6-BIO increased the inhibitory markers PD-L1 and PD-L2 (Supplementary Figure S1), while activation markers CD80, CD86 (Supplementary Figure S1), and the migration marker CCR7 (Figure 3A) were decreased. All of these changes followed a dose-dependent pattern.

ICG-001 at doses of 2 and 8 μM tended to decrease PD-L1, PD-L2, and CD86 (Supplementary Figure S1), and to increase CCR7 expression (Figure 3B). At doses of 2.5 and 10 μM, axitinib tended to decrease the inhibitory marker PD-L2, but not PD-L1, and additionally decreased CD86 (Supplementary Figure S1) and CCR7 expressions (Figure 3B). Nitazoxanide at doses of 5 and 20 μM tended to decrease PD-L2 expression (Supplementary Figure S1) but had limited effects on PD-L1, CD86 (Supplementary Figure S1), and CCR7 expressions (Figure 3B). Both axitinib and nitazoxanide downregulated PD-L2 compared to ICG-001. Again, considerable quantitative variation existed between the different healthy donors.

### 3.5. Effects of 6-BIO versus Potential β-Catenin Inhibitors on Migration of moDCs

The ability of cells to migrate towards a CCL19 concentration gradient was analyzed in the trans-well assay with CCL19 added to the medium in the lower chamber (Figure 3C). Neither ICG-001, nor axitinib, nor nitazoxanide affected the migration of LPS-matured DCs significantly, as exemplified in Figure 3C. A statistically significant increase in migration was found when ICG-001 and 6-BIO were combined (Figure 3C). Similar trends were found for the combination of either axitinib or nitazoxanide with 6-BIO (Figure 3C).

### 3.6. 6-BIO Decreased Antigen Uptake in Immature moDCs

**Figure 4:**
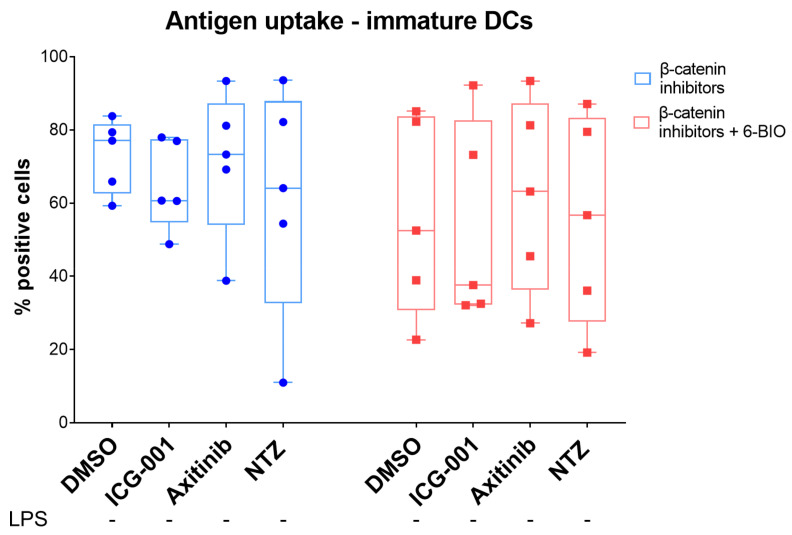
Antigen uptake assay. Immature moDCs obtained from healthy donors were treated with β-catenin inhibitors (4 µM ICG-001, 10 µM axitinib, 10 µM nitazoxanide (NTZ)) with or without 0.5 µM 6-BIO or DMSO (vehicle) for 24 h. Cells were left untreated with LPS as immature DCs. A total of 50,000 cells were incubated with FITC-dextran at 37 °C. After 1 h of incubation, cells were washed and analyzed immediately by flow cytometry. Each symbol represents a different donor, and lines represent the median.

Uptake of fluorescent dextran was used to evaluate the ability of moDCs to take up antigen. In order to examine the effect of β-catenin signaling on endocytic capacity, both immature (Figure 4) and mature (Supplementary Figure S2) moDCs were treated with ICG-001 (4 µM), axitinib (10 µM), and nitazoxanide (10 µM) with or without 6-BIO (0.5 µM) for 24 h. As shown in Supplementary Figure S2, FITC-dextran accumulated in immature moDCs, while matured moDCs showed clear reduction in FITC-dextran uptake. The addition of 6-BIO tended to result in less FITC-dextran uptake in comparison to the cells treated only with their respective β-catenin inhibitor. This pattern was more pronounced in immature moDCs. However, ICG-001 treatment also tended to reduce antigen uptake and did not compete with the 6-BIO-induced reduction of fluorescent dextran uptake. Clear stimulatory or inhibitory effects of axitinib or nitazoxanide were not found in this assay, with the possible exception that axitinib limited the 6-BIO-induced decrease of dextran uptake (Figure 4).

### 3.7. Compounds Tested in the Allogeneic Mixed Leukocyte Reaction (MLR)

**Figure 5:**
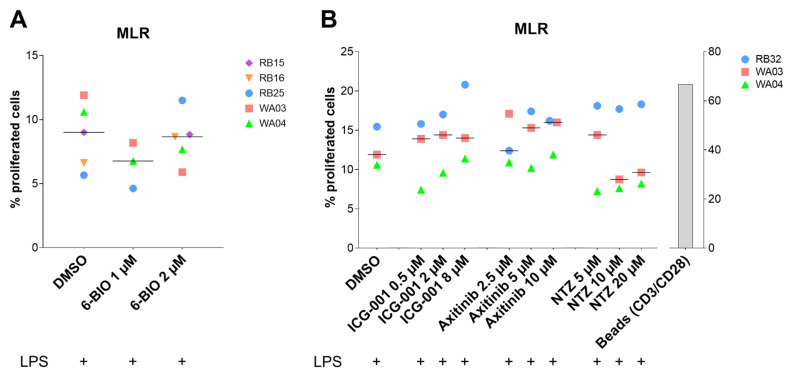
Mixed leukocyte reaction (MLR) performed between moDCs and monocyte-depleted PBMCs from another healthy donor. The moDCs were treated for 24 h with either indicated concentrations of 6-BIO or β-catenin inhibitors (type and concentrations as shown) and with concomitant LPS during the last 23 h. DMSO (vehicle) is the mature moDCs control. Allogeneic PBMCs were prelabeled with CFSE and cocultured with treated moDCs for 5 days. (**A**,**B**) Percentage of proliferated cells were measured by reduction in CFSE intensity. Each symbol indicates a different healthy donor, in 3 to 5 independent experiments. Donors were anonymized and each buffy coat labeled by initials of experimenter (RB or WA) and consecutive buffy coat number. Medians are indicated by bars.

To analyze the T cell stimulatory capacity of the cells, an allogeneic mixed leukocyte reaction (MLR) was performed. However, addition of the compounds did not have clear effects on the T cell stimulatory capacity in this assay (Figure 5).

### 3.8. Treatment of iDC with LPS Induced Extensive Changes in Gene Expression Pattern

Gene expression was analyzed using Illumina-based RNA-seq. Per sample, a median of 2.83 × 10^7^ (IQR 2.73 × 10^7^ to 2.91 × 10^7^) reads successfully mapped to the human genome and were available for further analysis, such as the identification of differentially expressed genes between the different treatment conditions. As expected, the most dramatic change in gene expression patterns was observed between the immature and mature dendritic cell populations, with more than 4400 differentially expressed genes (2049 upregulated genes with IQR_log2FC_ of 1.387 to 2.882, and 2364 downregulated genes with IQR_log2FC_ of −4.19 to −1.583) as a result of the maturation induced by LPS.

### 3.9. 6-BIO Induced the Expression of Genes Involved in the Wnt Signaling Pathway in LPS-Matured DC

**Figure 6:**
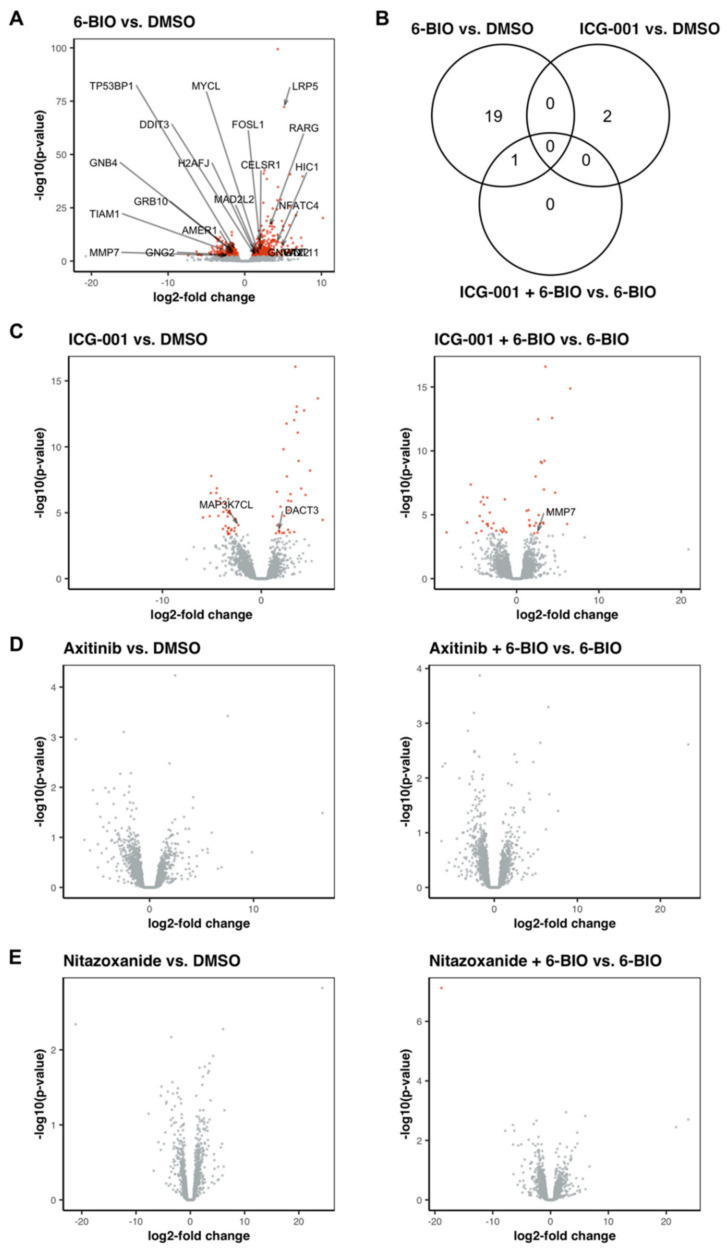
Volcano plots showing the changes in gene expression in LPS-matured dendritic cells (DC) following treatment with 6-BIO, ICG-001, axitinib, and nitazoxanide. Gene counts were acquired by RNA-seq, followed by differential gene expression (DGE) analysis. The x-axis is showing the gene expression changes as log_2_-fold change, while the y-axis is showing -log_10_(*p*-values) of the statistical test. (**A**,**C**–**E**) Differentially expressed genes following treatment of LPS-matured DC with either 6-BIO, ICG-001, axitinib, nitazoxanide, or a combination, as indicated. Genes with an adjusted *p*-value of <0.1 are shown in red. Gene symbols are added for genes associated with Wnt signaling pathways (KEGG pathway hsa04310, Reactome pathway R-HSA-195721, and GO biological term GO0016055). (**B**) Venn diagram showing the overlap of differentially expressed genes (adjusted *p*-value < 0.1) associated with Wnt signaling pathways between the different groups.

Treatment of mature dendritic cells with 6-BIO showed a total of 638 differentially expressed genes (402 upregulated genes, IQR_log2FC_ 1.751 to 3.29, and 236 downregulated genes, IQR_log2FC_ −2.479 to −1.377), representing around 3.7% of the gene set used for the DGE analysis. Genes involved in Wnt signaling pathways, such as the nuclear factor of activated T cells 4 (NFATC4), Wnt11, or LRP5, were identified among the most upregulated genes (Figure 6). Genes associated with biological processes such as cell migration, responses to stimulus, or signaling processes were enriched when supplying our list of upregulated genes (Supplementary Table S2). A similar observation was made when using the genes showing a downregulation following treatment with 6-BIO, pointing towards a major reorganization of the cellular signalosome (Supplementary Table S3).

### 3.10. Treatment with ICG-001 Alone or in Combination with 6-BIO Resulted in Changes in the Gene Expression Pattern of LPS-Matured DC, While Axitinib and Nitazoxanide Did Not Show Strong Effects on the Transcriptome of LPS-Matured DC

Treatment with axitinib alone failed to result in any significant changes in gene expression when compared with mature dendritic cells without axitinib treatment (Figure 6D). The same was observed for the treatment with nitazoxanide (Figure 6E). Treatment with ICG-001, on the other hand, resulted in 67 genes showing significant differences compared to mature dendritic cells without treatment (Figure 6C). Most of the 33 genes upregulated in response to the ICG-001 treatment (IQR_log2FC_ 2.117 to 3.588) are involved in biosynthesis and metabolic processes (Supplementary Table S4), while most of the 34 downregulated genes (IQR_log2FC_ −4.124 to −3.204) are involved in immune regulatory pathways (Supplementary Table S5).

Genes that have been shown to be influenced in their expression by the addition of ICG-001 include, for example, the genes S100A4 or BIRC5 (survivin) [5]. Both genes are reported to be downregulated in response to a treatment of SW480 colon carcinoma cells with 25 μM ICG-001. While we do observe the same gene expression pattern of downregulated S100A4 and BIRC5, their extent of downregulation in our LPS-matured DC culture does not reach a level of significance necessary for a definite conclusion (Supplementary Table S6).

When 6-BIO-treated mature DCs were additionally treated with ICG-001, 49 differentially expressed genes were found (25 upregulated genes, IQR_log2FC_ 2.158 to 3.389, and 24 downregulated genes, IQR_log2FC_ −4.298 to −2.34), compared to mature DCs treated only with 6-BIO. When focusing on either immune system or signal transduction processes, treatment of ICG-001 in the context of 6-BIO resulted in an upregulation of proteins involved in signal transduction pathways, while downregulated genes were mainly involved in immune system processes, as shown in Figure 6C. Without the addition of 6-BIO, ICG-001 treatment induced an upregulation of DACT3, a gene encoding the protein dishevelled binding antagonist of beta catenin 3. In combination with 6-BIO, ICG-001 showed a specific induction of MMP7, a target gene of β-catenin/TCF-4 signaling.

Treatment with axitinib or nitazoxanide in combination with 6-BIO did not result in any major changes of the transcriptome of LPS-matured DCs.

## 4. Discussion

Improved DCs are much needed for next-generation cancer immunotherapy [25,26,27]. One attractive possibility is that β-catenin signaling can be exploited to generate more robust and potent therapeutic DCs.

We have previously reported a β-catenin-responsive model system of moDCs derived from buffy coats of healthy blood donors [12]. Utilizing this model system in the present work, we aimed to investigate the ability of the two small molecular compounds axitinib and nitazoxanide to inhibit β-catenin compared with the β-catenin inhibitor ICG-001.

Initially, the toxicity of axitinib and nitazoxanide in moDC was tested using the MTS assay. A favorable toxicity profile was found for both axitinib and nitazoxanide, also compared with 6-BIO and ICG-001 at the doses used for the subsequent experimental studies. The low toxicity of axitinib is consistent with previously published work with this compound in moDCs [28]. We are not aware of any previous experimental publications on nitazoxanide in dendritic cells, although there are several reports suggesting stimulation of innate immunity and viral and parasitic defenses [19].

Next, we confirmed that our experimental model worked as intended and as previously published by showing that 6-BIO caused a dose-dependent accumulation of total β-catenin in mature DCs, and additionally in nuclear accumulation of β-catenin. A basal β-catenin activation that was dose-dependently augmented by the β-catenin stimulator 6-BIO and inhibited by the β-catenin inhibitor ICG-001 was demonstrated in moDCs matured by LPS [12]. One of the most pronounced effects was the ICG-001 dose-dependent increase in secreted pro-inflammatory IL-12p70, as also previously reported [12]. Interestingly, axitinib, in contrast to nitazoxanide, was able to induce increased secretion of IL-12p70, and this increase was counteracted by 6-BIO. Axitinib, again in contrast to nitazoxanide, additionally showed a trend to dose-dependent decrease of IL-10 secretion. This effect was less potent than the ICG-001 dose-dependent decrease of IL-10 secretion. However, axitinib was more potent in its stimulation of IL-12p70 secretion and inhibition of IL-10 secretion than the commercial β-catenin inhibitor XAV-939. XAV-939 reduces β-catenin activity by inhibition of tankyrase, an enzyme that degrades AXIN-2 of the β-catenin destruction complex in the cytoplasm [29]. It should be noted that a previous publication found discrepant results by showing that axitinib clearly reduced IL12-p70 in similar experiments with their moDCs [28]. Currently, we have no explanation for this difference. In our present study, magnetic bead-based kits were used for monocyte isolation, whereas the other group used plastic adherence. Additionally, considerable variation of quantitative cytokine secretion among moDCs derived from buffy coats of different healthy blood donors has been reported previously [12,30].

Next, treatment of moDCs with axitinib and nitazoxanide were compared to ICG-001 in several functional assays. The ability to migrate is important for DC function. One key mechanism of DC chemotaxis is driven by the ligands CCL21 and CCL19 upon binding to their receptor CCR7. In the trans-well assay, DCs are placed in the upper chamber and tested for their migration through a membrane to the lower chamber that contains a higher concentration of CCL19. The 6-BIO treatment reduced CCR7 surface expression of moDCs in contrast to ICG-001, which increased CCR7 expression. Axitinib and nitazoxanide treatment had limited effect on CCR7 expression, consistent with another study that did not find that axitinib reduced CCR7 expression [31]. It could be expected that the readout of the migration assay should reflect changes in CCR7 expression. However, this was not found in the present study. The increase in the migratory capacity of moDC after treatment with 6-BIO in combination with axitinib, nitazoxanide, and ICG-001 suggests a combinatorial effect of the tested compounds affecting signal transduction pathways other than beta-catenin. An independent publication reported a clear negative effect of axitinib on migration of moDCs [28].

Uptake of FITC-labeled dextran was used to assay the endocytic capability of moDCs. Consistent with our knowledge that antigen uptake is far more efficient in immature DCs than in mature DCs [32], LPS-induced maturation alone greatly reduced FITC-dextran uptake in mature, compared with immature, DCs. In immature moDCs, 6-BIO decreased fluorescent dextran uptake. ICG-001, axitinib, and nitazoxanide did not appear to have significant effects on FITC-dextran uptake in either mature or immature DCs, and no dose-dependent effects were evident. There might be some opposing effects between 6-BIO and axitinib, but our present conclusion is that the fluorescent uptake assay does not clearly reflect competitive effects on β-catenin signaling.

While cytokine secretion, activation state, or antigen uptake are important factors in defining the efficiency of dendritic cells, the ultimate task of dendritic cells is to activate T cells. In this study, an allogeneic mixed leukocyte reaction (MLR) was used to assess this crucial role of dendritic cells. We were not able, however, to find significant effects of the compounds at the tested concentrations on lymphocyte proliferation in this assay.

We cannot exclude the possibility that the functional assays used in this study may be affected by signal pathway cross-talks, thereby complicating their interpretation. Both β-catenin and ICG-001 are transcription factors and compete with each other within their common transcription complex [12]. Axitinib and nitazoxanide have been found to phenocopy each other regarding β-catenin inhibition in colon cancer cells, but with different molecular targets and mechanisms [15,17]. Whereas axitinib targeted the ubiquitin ligase SHPRH to increase ubiquitination and proteasome-mediated degradation of β-catenin [15], nitazoxanide targeted PADI2 to increase citrullination and degradation of β-catenin [17] in colon cancer cells. Because these small molecular compounds have previously shown effects at the final steps of the signal transduction pathway in the nucleus of different cell types, we anticipated that gene expression analyses could be useful to analyze their effect on β-catenin transcriptional regulation. Different effects of small molecular compounds in tumor cells versus dendritic cells have been observed in other settings, e.g., for the STAT3 inhibitor stattic [33].

Extensive gene expression changes were observed during LPS-maturation of iDCs, as we have also published previously [12]. Cotreatment with 6-BIO caused hundreds of genes to become either up- or downregulated in LPS-matured moDCs. The relevance of β-catenin signaling in this system was indicated by the WNT/β-catenin-associated genes that were among the most upregulated in 6-BIO-treated moDCs. Treatment of mature moDCs using ICG-001 caused a number of significant changes of gene expression, several of which were reciprocal to 6-BIO induced changes, as we have also found in previous experiments [12]. In contrast, at the chosen significance threshold, neither axitinib nor nitazoxanide affected gene expression patterns in mature moDCs. A specific examination of the small molecular compounds’ impact on putative β-catenin signaling was achieved by cotreatment of mature moDCs with 6-BIO plus either ICG-001 or axitinib or nitazoxanide. Gene expression changes were examined between cotreated DCs and DCs treated with 6-BIO only. Again, only ICG-001 treatment, in contrast to either axitinib or nitazoxanide, caused substantial gene expression changes above the chosen significance thresholds.

Both stimulatory [34] and inhibitory activity [28] of axitinib in DCs have been published. Axitinib has been reported to enhance immunotherapeutic vaccines in mice [34,35] and humans [36]. According to one report, the addition of axitinib during the LPS maturation step of human moDCs resulted in impaired expression of activation markers and costimulatory molecules CD80, CD83, and CD86, and the migration marker CCR7, decreased secretion of IL-12p70, impaired induction of allogeneic T cell proliferation in the MLR, and inhibited DC migration [28]. Additional works have found that VEGFR inhibitors may adversely affect migration of DCs [37]. In our present work, we found that axitinib reduced CD86 and CCR7 membrane expression with little effect on CD80 and CD83.

Axitinib is one of several tyrosine kinase inhibitors that were approved as antiangiogenic agents by predominantly targeting the VEGF receptor (VEGFR) [38]. Subsequent combination of axitinib or other VEGFR inhibitors with immune checkpoint inhibition has improved therapy of invasive kidney cancer substantially. Synergy is suggested by the combination, yet the mechanisms of interaction are not fully uncovered [38]. VEGF has been found to inhibit dendritic cell maturation and other immune cells via different pathways [38,39,40]. Interestingly, the tyrosine kinase inhibitor, pazopanib, used clinically on similar indications as axitinib to inhibit VEGFRs and other tyrosine kinases, was found to inhibit β-catenin signaling and thereby increased dendritic cell maturation markers, CCR7, and decreased IL-10 secretion and endocytosis [41]. The tyrosine kinase inhibitors sorafenib and sunitinib, also used on similar clinical indications, affected dendritic cell function differentially [37]. Axitinib inhibits additional tyrosine kinases than VEGFRs [18] and impinges on different pathways that converge on other transcription factors than β-catenin, such as STAT3 [38]. It is therefore understandable that it is problematic to dissect any isolated effect of axitinib mediated on DC function via β-catenin.

Similar challenges apply to nitazoxanide, which has been reported to interfere with multiple signaling mechanisms in different cell types [19], including β-catenin signal inhibition [17,19,42,43,44].

## 5. Conclusions

Both axitinib and nitazoxanide have the ability to affect moDCs at doses that are not toxic. In particular, stimulation of IL-12p70, as demonstrated by axitinib, is noteworthy. The final conclusion is, however, that neither axitinib nor nitazoxanide was able to regulate β-catenin signaling of moDCs, in contrast to, and in comparison with, ICG-001 and 6-BIO. These effects are valuable to take into consideration in the development of cancer combination therapy. Furthermore, this work validated the present model system in order to test or screen the potential of new β-catenin inhibitors in moDCs.

## Data Availability

The present study does not report any individual patient data. Only anonymized patient samples have been used. The RNA-seq data presented in this study is openly available in the ArrayExpress database at EMBL-EBI (www.ebi.ac.uk/arrayexpress (accessed on 13 July 2021)) under accession number E-MTAB-10757.

## Supplementary Materials

The following are available online at https://www.mdpi.com/article/10.3390/biomedicines9080949/s1, Figure S1: moDCs obtained from healthy donors were treated with indicated concentration of 6-BIO or β-catenin inhibitors (type and concentrations as shown) for 24 h, Figure S2: Antigen uptake assay, Figure S3: Representative gating strategy for flow cytometry analysis, Table S1: ELISA IL-12 p70 concentrations (values in pg/ml) for Figure 2, Table S2: GO term enrichment analysis for dif-ferentially expressed genes of the comparison mDC + 6-BIO vs. mDC + DMSO (for up-regulated genes only), Table S3: GO term enrichment analysis for differentially expressed genes of the com-parison mDC + 6-BIO vs. mDC + DMSO (for down-regulated genes only), Table S4: GO term en-richment analysis for differentially expressed genes of the comparison mDC + ICG-001 vs. mDC + DMSO (for up-regulated genes only), Table S5: GO term enrichment analysis for differentially ex-pressed genes of the comparison mDC + ICG-001 vs. mDC + DMSO (for down-regulated genes only), Table S6: Genes effected by ICG-001 treatment and their representation in the dataset.
