# Supplementary material for: Evaluation of β-Catenin Inhibition of Axitinib and Nitazoxanide in Human Monocyte-Derived Dendritic Cells
Source: Biomedicines. 2021 Aug 3;9(8):949. doi: 10.3390/biomedicines9080949 (PMC8391762; doi:10.3390/biomedicines9080949)
Supplement: Supplementary file 1 [file biomedicines-09-00949-s001.zip › biomedicines-1317980-supplementary.pdf]

Supplementary Materials

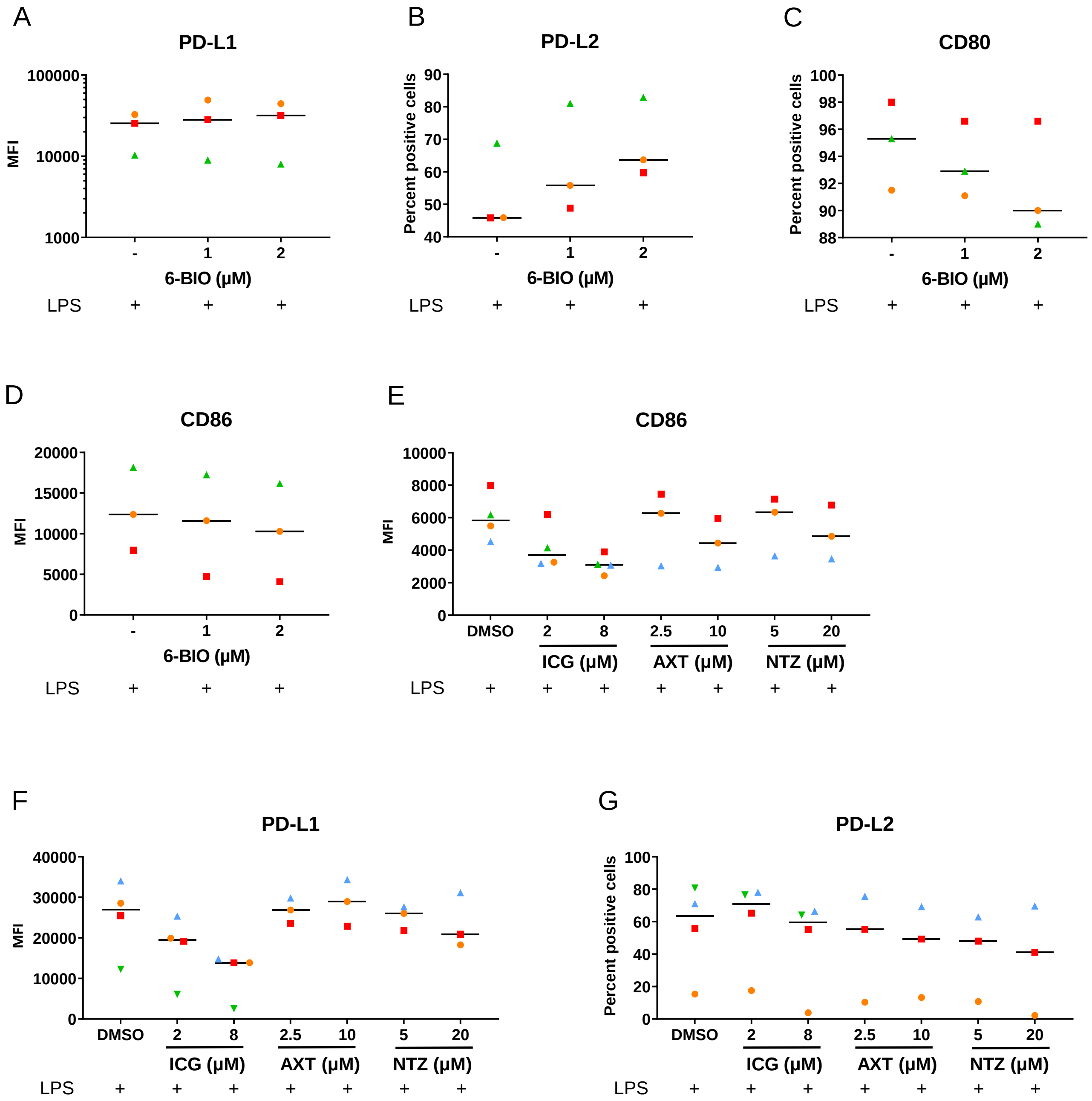

Supplementary Figure 1. moDCs obtained from healthy donors were treated with indicated concentration of 6-BIO or β-catenin inhibitors (type and concentrations as shown) for 24 hours. DMSO (vehicle) served as mature moDCs control and cells were treated with LPS for the last 23 hours. A - G) cell surface marker was measured with flow cytometry and shown as change of percentage of positive cells or median fluorescence intensity (MFI). Each symbol represents a different donor and lines represent the median.

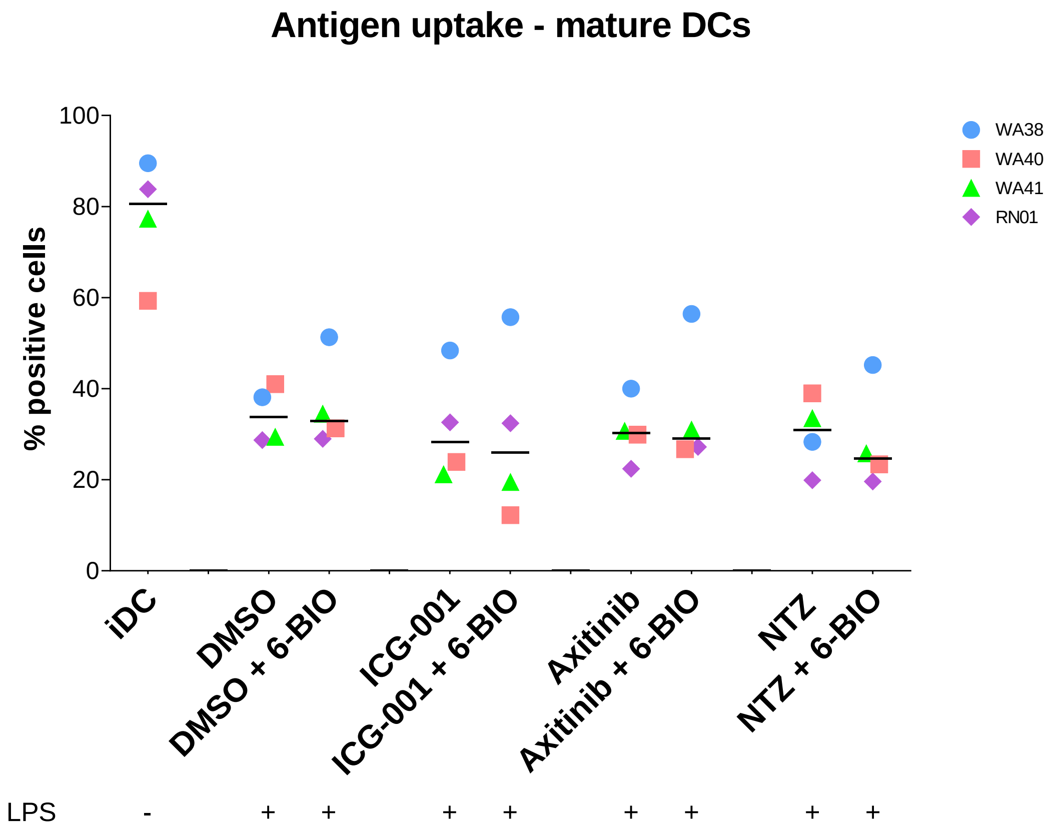

Supplementary Figure 2. **Antigen uptake assay.** moDCs obtained from healthy donors were treated with indicated β-catenin inhibitors (ICG-001 4 μM, axitinib 10 μM, nitazoxanide (NTZ) 10 μM) with/without 6-BIO 0.5 μM or DMSO (vehicle) for 24 hours. mature-DCs, treated with 100 ng/ml LPS for the last 23 hours except iDCs. 50,000 cells were incubated with FITC-dextran at 37°C. After 1 hour of incubation, cells were washed and analyzed immediately by flow cytometry. Each symbol represents a different donor and lines represent the median.

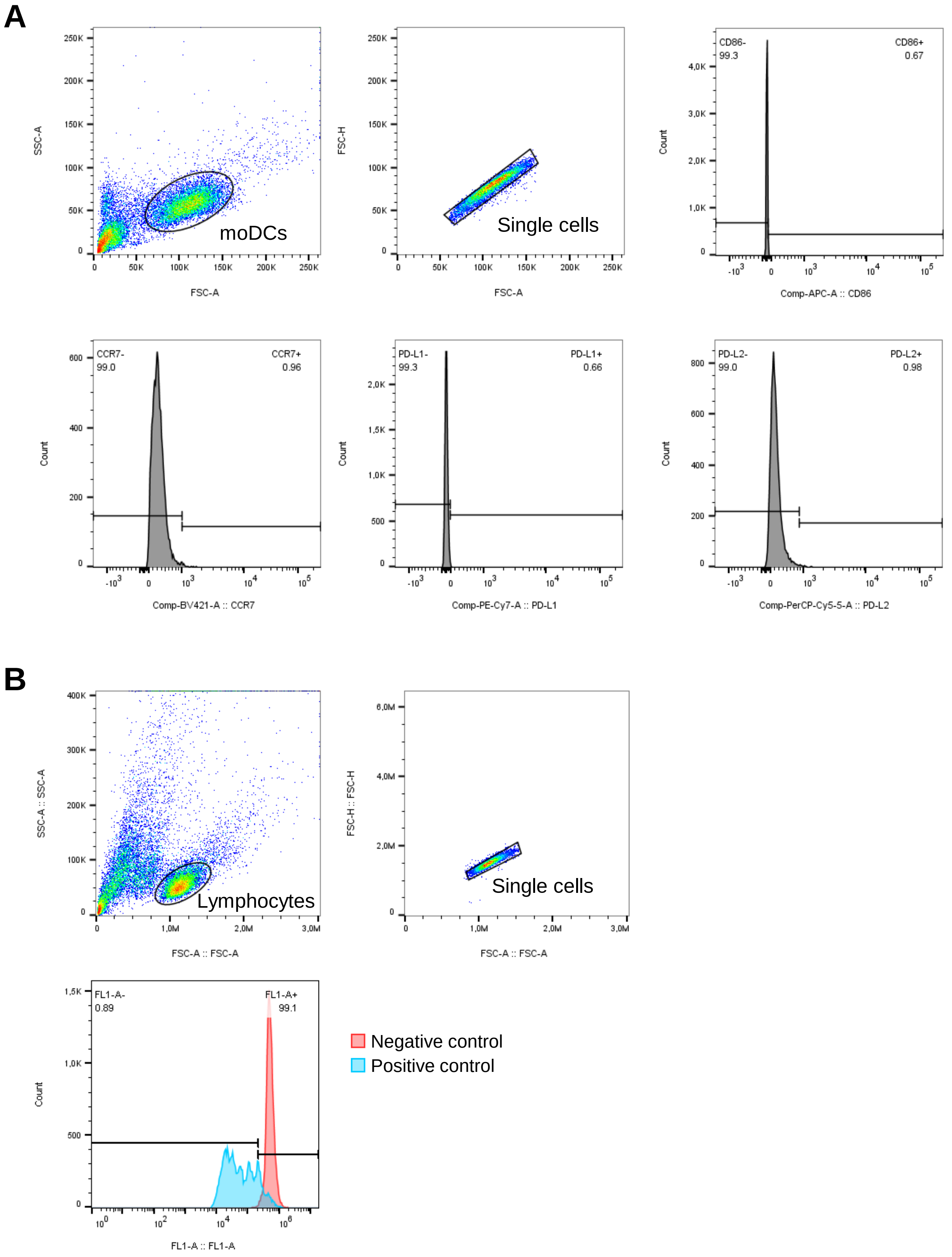

Supplementary Figure 3. **Representative gating strategy for flow cytometry analysis.** A) For moDCs surface markers, unstained samples were used to set the gates. B) For Mixed Leukocyte Reaction (MLR), negative controls were used to set the gates. 1% false-positive events were accepted throughout the analysis.

Supplementary Table 1A. ELISA IL-12 p70 concentrations (values in pg/ml) for figure 2C.

	Donor RB21 (pg/ml)	Donor RB25 (pg/ml)	Donor WA03 (pg/ml)	Donor WA04 (pg/ml)	Donor WA05 (pg/ml)
DMSO	2395	468	1591	14991	19271
6-BIO 1 µM	315	364	88	27.406	103
6-BIO 2 µM	160	56	54.5	5.864	116

Supplementary Table 1B. ELISA IL-10 concentrations (values in pg/ml) for figure 2D.

	Donor RB21 (pg/ml)	Donor RB25 (pg/ml)	Donor WA03 (pg/ml)	Donor WA04 (pg/ml)	Donor WA05 (pg/ml)
DMSO	441	26	9246	3353	2744
6-BIO 1 µM	1398	182	14889	3340	2524
6-BIO 2 µM	917	148	11100	2100	2939

Supplementary Table 1C. ELISA IL-12 p70 concentrations (values in pg/ml) for figure 2E.

	Donor RB32 (pg/ml)	Donor RB33 (pg/ml)	Donor RB34 (pg/ml)	Donor WA03 (pg/ml)	Donor WA04 (pg/ml)	Donor WA05 (pg/ml)
DMSO	1528	23000	625	1375	14991	19271
ICG-001 0.5 µM	2195			866	15027.7	15027
ICG-001 2 µM	3760			1516	20578.87	39790
ICG-001 8 µM	13722			6173	49906.9	45661
Axitinib 2.5 µM	1487	46200	2725			
Axitinib 5 µM	3637	46400	1908			
Axitinib 10 µM	3741	24600	2430			
NTZ 5 µM	963	15000	821			
NTZ 10 µM	1141	12400	905			
NTZ 20 µM	627	16000	641			

Supplementary Table 1D. ELISA IL-10 concentrations (values in pg/ml) for figure 2F.

	Donor RB32 (pg/ml)	Donor RB33 (pg/ml)	Donor RB34 (pg/ml)	Donor WA03 (pg/ml)	Donor WA04 (pg/ml)	Donor WA05 (pg/ml)
DMSO	1219	105	712	9246	3353	2782
ICG-001 0.5 µM	516			8746	444	1461
ICG-001 2 µM	85			4696	129	718
ICG-001 8 µM	42			1322	74	96
Axitinib 2.5 µM	677	239	1096			
Axitinib 5 µM	1325	155	836			
Axitinib 10 µM	1378	25	606			
NTZ 5 µM	1076	102	625			
NTZ 10 µM	323	75	751			
NTZ 20 µM	139	100	686			

Supplementary Table 1E. ELISA IL-12 p70 concentrations (values in pg/ml) for figure 2G.

	Donor WA31 (pg/ml)	Donor WA32 (pg/ml)	Donor WA33 (pg/ml)	Donor WA34 (pg/ml)	Donor WA51 (pg/ml)	Donor WA53 (pg/ml)	Donor WA54 (pg/ml)	Donor WA55 (pg/ml)
DMSO	3566.35	402.15	2115.37	39225.6	86.493	298.75	316.6	105.45
6-BIO 0.5 µM	463.45	5.32	324.495	1319.6	30.995	27.2	36.35	37.95
ICG-001 4µM	2492	1573.83	2226.135	30483	209.649	245.05	417.3	136.45
ICG-001 + 6-BIO	1058.25	91.82	1126.36	4566.6	47.884	43.45	56.6	50.1
Axitinib 10µM	5766.05	2926.61	2450.59	78156	269.45	244	937.85	753.2
Axitinib + 6-BIO	946.4	266.56	1961.06	11215.4	108.858	49.95	107.05	114.8
NTZ 10µM	3842.85	541.73	2283.105	21500.4	29.825	116.873	259.15	146.1
NTZ + 6-BIO	567.85	5.48	410.94	977.7	17.948	8.822	35.95	55.25
XAV-939 10µM	1360.35	187.45	2492.875	7125.7	19.431	19.418	61.65	58.05
XAV-939 + 6-BIO	577.3	2.5	358.1	745.3	27.293	6.487	56.9	59.3

Supplementary Table 1F. ELISA IL-10 concentrations (values in pg/ml) for figure 2H.

	Donor WA31 (pg/ml)	Donor WA32 (pg/ml)	Donor WA33 (pg/ml)	Donor WA34 (pg/ml)	Donor WA51 (pg/ml)	Donor WA53 (pg/ml)	Donor WA54 (pg/ml)	Donor WA55 (pg/ml)	Donor WA49 (pg/ml)
DMSO	290.6	362.65	2568.325	1711.7	5.739	379.3	340.45	167.95	367.85
6-BIO 0.5 µM	1492.25	856.65	3762.15	2523.55	36.171	761.5	1019.8	400.85	366.25
ICG-001 4µM	59.65	35.367	402.15	77.302	4.045	30.081	48.65	23.6	142.35
ICG-001 + 6-BIO	277.8	171.65	1474.225	303.232	11.768	96.952	182.85	73.7	175.4
Axitinib 10µM	159.8	316.3	2946.7	3275.65	4.865	166.65	213.35	51.05	152.55
Axitinib + 6-BIO	685.2	1008.25	6628.7	3511.55	15.616	385.35	576.8	201.85	139.3
NTZ 10µM	249.35	599	2838.7	1358.2	4.858	202.5	315.75	125.55	434.15
NTZ + 6-BIO	509.55	1064.7	4798.475	2040.3	27.82	490.4	875.85	309.95	400.15
XAV-939 10µM	462.9	655.2	2525.8	795.2	19.47	428.7	637.8	167.05	393.8
XAV-939 + 6-BIO	899.9	1049.6	4380.125	2160.25	32.397	647.8	935.45	276.6	398.52

Supplementary Table 2. GO term enrichment analysis for differentially expressed genes of the comparison mDC + 6-BIO vs. mDC + DMSO (for up-regulated genes only). The table shows GO identifiers in combination with GO terms, as well as the number of annotated genes in the GO term gene set (Annot.).

GO ID	*GO Term*	*Annot.*	*S*	*Expect.*	*Fisher*
GO:0050896	response to stimulus	6449	210	154.9200	4.6000e-11
GO:0032501	multicellular organismal process	5014	172	120.4500	9.1800e-10
GO:0016477	cell migration	1144	60	27.4800	4.4420e-09
GO:0042221	response to chemical	3288	123	78.9900	1.6098e-08
GO:0048870	cell motility	1247	62	29.9600	1.7896e-08
GO:0051674	localization of cell	1247	62	29.9600	1.7896e-08
GO:0023052	signaling	4532	155	108.8700	2.7086e-08
GO:0040011	locomotion	1368	65	32.8600	4.2858e-08
GO:0007154	cell communication	4560	155	109.5500	4.3151e-08
GO:0006928	movement of cell or subcellular componen...	1577	71	37.8800	7.6166e-08
GO:0051271	negative regulation of cellular componen...	249	22	5.9800	1.4358e-07
GO:0051239	regulation of multicellular organismal p...	2371	94	56.9600	1.5107e-07
GO:0048513	animal organ development	2476	97	59.4800	1.5403e-07
GO:0040012	regulation of locomotion	767	43	18.4300	1.5964e-07
GO:0030855	epithelial cell differentiation	432	30	10.3800	1.7264e-07
GO:2000145	regulation of cell motility	742	42	17.8300	1.7445e-07
GO:0030336	negative regulation of cell migration	215	20	5.1600	2.3455e-07
GO:0007186	G protein-coupled receptor signaling pat...	538	34	12.9200	2.3713e-07
GO:0030334	regulation of cell migration	702	40	16.8600	2.9872e-07
GO:0001944	vasculature development	551	34	13.2400	4.1402e-07
GO:0072358	cardiovascular system development	551	34	13.2400	4.1402e-07

Supplementary Table 3. GO term enrichment analysis for differentially expressed genes of the comparison mDC + 6-BIO vs. mDC + DMSO (for down-regulated genes only). The table shows GO identifiers in combination with GO terms, as well as the number of annotated genes in the GO term gene set (Annot.).

GO ID	*GO Term*	*Annot.*	*S*	*Expect.*	*Fisher*
GO:0051716	cellular response to stimulus	5389	120	84.5800	3.3000e-07
GO:0070887	cellular response to chemical stimulus	2544	69	39.9300	1.0400e-06
GO:0048518	positive regulation of biological proces...	4785	108	75.1000	1.6200e-06
GO:1901700	response to oxygen-containing compound	1276	42	20.0300	2.7700e-06
GO:0051480	regulation of cytosolic calcium ion conc...	226	15	3.5500	2.8900e-06
GO:0071310	cellular response to organic substance	2100	59	32.9600	3.0300e-06
GO:0007154	cell communication	4560	103	71.5700	3.8800e-06
GO:0023052	signaling	4532	102	71.1300	5.5400e-06
GO:0007204	positive regulation of cytosolic calcium...	209	14	3.2800	5.6200e-06
GO:0035556	intracellular signal transduction	2258	61	35.4400	7.0000e-06
GO:1901701	cellular response to oxygen-containing c...	922	33	14.4700	7.1200e-06
GO:0048522	positive regulation of cellular process	4388	98	68.8700	1.5560e-05
GO:0010033	response to organic substance	2533	65	39.7600	1.7160e-05
GO:0050794	regulation of cellular process	8051	154	126.3600	1.9100e-05
GO:0042832	defense response to protozoan	22	5	0.3500	1.9660e-05
GO:0034097	response to cytokine	1017	34	15.9600	2.1510e-05
GO:0001562	response to protozoan	23	5	0.3600	2.4800e-05
GO:0071345	cellular response to cytokine stimulus	941	32	14.7700	2.7940e-05
GO:0050896	response to stimulus	6449	130	101.2200	2.8210e-05
GO:0042221	response to chemical	3288	78	51.6100	2.9130e-05

Supplementary Table 4. GO term enrichment analysis for differentially expressed genes of the comparison mDC + ICG-001 vs. mDC + DMSO (for up-regulated genes only). The table shows GO identifiers in combination with GO terms, as well as the number of annotated genes in the GO term gene set (Annot.).

GO ID	*GO Term*	*Annot.*	*S*	*Expect.*	*Fisher*
GO:1901607	alpha-amino acid biosynthetic process	56	5	0.1400	2.8000e-07
GO:0008652	cellular amino acid biosynthetic process	68	5	0.1700	7.4000e-07
GO:0006563	L-serine metabolic process	11	3	0.0300	2.4900e-06
GO:0009070	serine family amino acid biosynthetic pr...	15	3	0.0400	6.8100e-06
GO:0017144	drug metabolic process	446	8	1.1400	1.2280e-05
GO:0071549	cellular response to dexamethasone stimu...	24	3	0.0600	2.9820e-05
GO:1901605	alpha-amino acid metabolic process	152	5	0.3900	3.8630e-05
GO:0097327	response to antineoplastic agent	80	4	0.2000	4.8990e-05
GO:0046394	carboxylic acid biosynthetic process	270	6	0.6900	5.4570e-05
GO:0016053	organic acid biosynthetic process	271	6	0.6900	5.5700e-05
GO:0009069	serine family amino acid metabolic proce...	30	3	0.0800	5.9200e-05
GO:0071548	response to dexamethasone	31	3	0.0800	6.5430e-05
GO:0044283	small molecule biosynthetic process	569	8	1.4600	7.0430e-05
GO:0009066	aspartate family amino acid metabolic pr...	38	3	0.1000	1.2000e-04
GO:0071385	cellular response to glucocorticoid stim...	42	3	0.1100	1.6000e-04
GO:0071384	cellular response to corticosteroid stim...	44	3	0.1100	1.9000e-04
GO:0071466	cellular response to xenobiotic stimulus	117	4	0.3000	2.1000e-04
GO:0044282	small molecule catabolic process	366	6	0.9400	2.9000e-04
GO:0009064	glutamine family amino acid metabolic pr...	53	3	0.1400	3.3000e-04
GO:0042446	hormone biosynthetic process	54	3	0.1400	3.5000e-04

Supplementary Table 5. GO term enrichment analysis for differentially expressed genes of the comparison mDC + ICG-001 vs. mDC + DMSO (for down-regulated genes only). The table shows GO identifiers in combination with GO terms, as well as the number of annotated genes in the GO term gene set (Annot.).

GO ID	*GO Term*	*Annot.*	*S*	*Expect.*	*Fisher*
GO:0019730	antimicrobial humoral response	55	8	0.1400	6.1000e-13
GO:0006952	defense response	1265	19	3.1400	4.8300e-12
GO:0006954	inflammatory response	601	14	1.4900	3.8240e-11
GO:0030593	neutrophil chemotaxis	91	8	0.2300	3.9970e-11
GO:0061844	antimicrobial humoral immune response me...	29	6	0.0700	6.3900e-11
GO:0071621	granulocyte chemotaxis	105	8	0.2600	1.2813e-10
GO:1990266	neutrophil migration	105	8	0.2600	1.2813e-10
GO:0032101	regulation of response to external stimu...	819	15	2.0300	1.7575e-10
GO:0009605	response to external stimulus	2094	21	5.2000	3.7610e-10
GO:0097530	granulocyte migration	122	8	0.3000	4.3026e-10
GO:0006955	immune response	1644	19	4.0800	4.9131e-10
GO:0006959	humoral immune response	147	8	0.3600	1.9102e-09
GO:0006950	response to stress	3236	24	8.0300	2.8453e-09
GO:0002376	immune system process	2414	21	5.9900	5.6275e-09
GO:0097529	myeloid leukocyte migration	182	8	0.4500	1.0337e-08
GO:0051707	response to other organism	1123	15	2.7900	1.4005e-08
GO:0043207	response to external biotic stimulus	1124	15	2.7900	1.4176e-08
GO:0030595	leukocyte chemotaxis	193	8	0.4800	1.6380e-08
GO:0009607	response to biotic stimulus	1151	15	2.8600	1.9581e-08
GO:0098542	defense response to other organism	853	13	2.1200	4.1847e-08

Supplementary Table 6. Genes effected by ICG-001 treatment and their representation in the dataset. Genes were manually selected based on the references shown. Statistical values for the comparison of LPS-matured DC + ICG-001 vs. LPS-matured DC + DMSO are shown.

HGNC symbol	*Ensembl ID*	*log2FC*	*p-value*	*adj. p-value*	*Reference*
BIRC5	ENSG00000089685	-0.7157	0.7074	1	Emami et al. 2004; Chan et al. 2015
CCND1	ENSG00000110092	0.8013	0.5697	1	Emami et al. 2004
CD44	ENSG00000026508	-0.1965	1.0000	1	Chan et al. 2015
CDH1	ENSG00000039068	0.1363	1.0000	1	Chan et al. 2015
EZH2	ENSG00000106462	-0.1493	1.0000	1	Chan et al. 2015
FOXM1	ENSG00000111206	-0.3174	1.0000	1	Chan et al. 2015
S100A4	ENSG00000196154	-1.1493	0.0372	1	Emami et al. 2004
VIM	ENSG00000026025	-0.4712	1.0000	1	Chan et al. 2015
